# Complement C5a‐triggered differentiated HL‐60 stimulates migration of THP‐1 monocytic leukocytes via secretion of CCL2

**DOI:** 10.1002/2211-5463.13144

**Published:** 2021-04-03

**Authors:** Syed Masudur Rahman Dewan, Mizuko Osaka, Michiyo Deushi, Masayuki Yoshida

**Affiliations:** ^1^ Department of Life Sciences and Bioethics Graduate School of Medical and Dental Sciences Tokyo Medical and Dental University Japan; ^2^ Department of Nutrition in Cardiovascular Disease Graduate School of Medical and Dental Sciences Tokyo Medical and Dental University Japan

**Keywords:** C5a, CCL2, chemotaxis, neutrophil‐like dHL‐60, monocytic leukocyte THP‐1, NF‐κB p65

## Abstract

Leukocytes play an important role in vascular inflammation prior to atherosclerosis. In particular, monocyte adhesion and migration to the endothelium contribute to the development of vascular inflammation. Previously, we showed the importance of neutrophils and complement C5a in the early phase of vascular inflammation in mice fed a high‐fat diet. However, the relationship between monocytes and neutrophils is not well understood. In this study, we elucidated the involvement of neutrophils in the migration of monocytes. We observed that C5a induces CCL2 expression in neutrophil‐like dHL‐60 cells. To investigate the physiological significance of CCL2 secretion, we performed a chemotaxis assay. Interestingly, dHL‐60 culture supernatant in the presence of C5a enhanced the migration of THP‐1 in comparison with the absence of C5a. Furthermore, CCL2 expression and secretion significantly increased in C5a‐stimulated dHL‐60 through the phosphorylation of NF‐κB p65. Actin polymerization on THP‐1 was enhanced by the presence of C5a compared with the absence of C5a when stimulated by a dHL‐60‐cultured medium. These results suggest that crosstalk between neutrophils and monocytes via CCL2 may play an important role in vascular inflammation.

## Graphical abstract

In this study, we found that neutrophil‐like dHL‐60 stimulated with C5a induces the migration of monocytic THP‐1 through phosphorylation of p65 NF‐κB and that dHL‐60‐derived CCL2 plays an important role in the crosstalk between these cells. These results suggest that there may be a transition process from acute to chronic inflammation in atherosclerotic vascular inflammation.

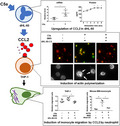

**Abbreviations**
CCL2: C‐C motif ligand 2
CCR2: C‐C motif receptor 2
dHL‐60: differentiated human promyelocytic cell line
dHL‐60‐CS: culture supernatant of dHL‐60
ELISA: enzyme‐linked immunosorbent assay
ERK: extracellular signal‐regulated kinase
HFD: high‐fat diet
MAPK: mitogen‐activated protein kinase
Neut‐CS: culture supernatant of mouse BM neutrophil
NF‐κB: nuclear factor‐kappa B
THP‐1: human monocytic leukemia cell line

Atherosclerosis is an inflammatory disease characterized by leukocyte adhesion, migration, and infiltration into an injured vascular endothelium [1]. In particular, a number of studies have addressed the role of monocytes in atherosclerosis. Monocytes have been shown to migrate under injured intima by lipid accumulation followed by differentiation into macrophages and form cells that progress vascular inflammation and atherosclerosis [2]. Recently, we showed the importance of neutrophils in a high‐fat diet‐induced vascular inflammation [3].

Neutrophils are first responders for infection or injury and protect the body against pathogens or stimuli [4]. Neutrophils have been shown to migrate to the inflammatory site by a gradient in the concentration of chemoattractant reagents, such as CXCL1 or C5a, prior to chronic inflammation or tissue repair [5]. Furthermore, neutrophils have been shown to crosstalk with other leukocyte subsets to regulate the immune system. B cells are regulated by the B‐cell‐activating factor cytokines produced by neutrophils [6], and the activation and the proliferation of T cells are suppressed by arginase 1 in neutrophils [7]. Moreover, neutrophils are involved in the polarization of macrophage through secretion of neutrophil gelatinase‐associated lipocalin in acute myocardial infarction [8]. However, the role of crosstalk between neutrophils and monocytes in atherosclerosis‐related vascular inflammation remains unknown. In a previous report, we indicated that complement C5a increased in mice fed a high‐fat diet (HFD), which induced a neutrophil adhesion on the femoral artery of mice. In addition, CCL2 was upregulated in the blood and circulating neutrophils of those mice [3]. However, the mechanism of monocyte migration by C5a‐stimulated neutrophils is unknown.

We, therefore, aim to prove that CCL2 secreted from neutrophil‐like differentiated HL‐60 stimulated by C5a induces the recruitment of monocytic leukocyte THP‐1 *in vitro*. Furthermore, we show that in THP‐1, actin polymerization is enhanced by dHL‐60‐cultured medium in the presence of C5a in this study.

## Materials and methods

### Cell cultures

Human monocytic leukemia cell line (THP‐1) was obtained from RIKEN BioResource Research Center Cell Bank (Tsukuba, Japan) and was maintained in RPMI‐1640 (FUJIFILM Wako Pure Chemical Corporation, Osaka, Japan) culture medium containing 10% fetal bovine serum (FBS), 100 IU/ml penicillin, and 0.17 mmol/L streptomycin (Gibco, Life Technologies Japan, Tokyo, Japan). The human leukemia cell line HL‐60 (ATCC) was cultured in RPMI‐1640 containing 10% FBS and penicillin/streptomycin. To differentiate HL‐60 cells, 1.3% dimethyl sulfoxide (DMSO; FUJIFILM Wako Pure Chemical Corporation) was added to the culture medium, and they were cultured for 5 days in an incubator maintained at 37°C with 5% CO_2_ [3, 9].

Differentiated HL‐60 (dHL‐60, 1 × 10^6^ cells) cells were stimulated by 3 nm recombinant human C5a (PeproTech Inc. NJ, USA) followed by each experiment.

### Treatment of dHL‐60 cells with IκB inhibitor

dHL‐60 (1 × 10^6^ cells) cells treated with C5a for 2 h were stimulated with or without 5 µm of the IκB inhibitor, BAY 11‐7082 (BAY; Merck KGaA), for 15 min followed by qRT‐PCR for CCL2.

### Quantitative RT‐PCR

mRNA was isolated from dHL‐60 cells stimulated with or without C5a by FastGene RNA Basic Kit (Nippon Genetics Co., Ltd. Tokyo, Japan). cDNA was synthesized by PrimeScript RT Master Mix (Takara Bio Inc. Shiga, Japan), and a quantitative RT‐PCR analysis using Thermal Cycler Dice Real Time System (Takara Bio Inc.) was performed to examine the CCL2 expression level. The relative expression levels of CCL2 were calculated by using ∆∆Ct methods and were normalized against ribosomal 18s (the internal control). The primers used in the experiment were human CCL2 forward: 5’‐CCAAGCAGAAGTGGGTTCAG‐3’ and reverse: 5’‐CTTGGGTTGTGGAGTGAGTG‐3’, and 18S ribosomal RNA forward: 5’‐GTAACCCGTTGAACCCCATT‐3’ and reverse 5’‐CCATCCAATCGGTAGTAGCG‐3’.

### ELISA

10^6^ cells of dHL‐60 cells were stimulated with 3nM C5a for 1, 2, 4, 6, or 8 hours. The CCL2 level in each culture supernatant was measured using anti‐human CCL2 antibody (BioLegend) by ELISA, as previously reported [3]. The absorbance at 450 nm was measured, and the concentration in the samples was calculated using standard curves (ARVO^TM^ X3 Multilabel Reader, Perkin Elmer).

### Western blotting

dHL‐60 cells (1 × 10^6^ cells) were stimulated with 3nM C5a for 15, 30, and 120 min. dHL‐60 cells were lysed with RIPA buffer containing protease and phosphatase inhibitors. Next, the protein concentration was measured using DC^TM^ Protein Assay (Bio‐Rad, Hercules, CA). Then, 10 µg of each protein was applied to 10% polyacrylamide gel, and the proteins were transferred to a polyvinylidene fluoride (PVDF) membrane (GE Healthcare UK Ltd.). Membranes were reacted with anti‐phospho (p)‐NF‐κB p65, NF‐κB p65, p‐ERK1/2, ERK1/2, p‐p38 MAPK, and p38 MAPK (Santa Cruz Biotechnology) following anti‐mouse or anti‐rabbit secondary antibody conjugated with horseradish peroxidase (GE Healthcare UK Ltd.). Immunoreactive proteins were detected with a luminol‐based enhanced chemiluminescence kit (Thermo Scientific). The signals were detected by LAS‐1000 (Fujifilm) and measured using Multi Gauge Software version 3.0 (Fujifilm). The relative expression of phosphorylated proteins was calculated by using 1.00 for the expression level of total proteins.

### Isolation of BM primary neutrophil and CD115‐positive primary monocyte from mouse

C57BL/6J mice (seven‐week‐old male) were purchased from Charles River Laboratories Japan, Inc. The experiments adhered to the APS Guiding Principles in the Care and Use of Animals and were approved by the Ethical Committee for Animal Experimentation of Tokyo Medical and Dental University. Bone marrow‐derived (BM‐) neutrophils were isolated from femurs and tibias of mice using anti‐Ly‐6G MicroBeads (Miltenyi Biotec) according to manufacturer’s protocol. CD115‐positive monocytes in flow‐through fraction were used as monocyte (Fig. S1).

Isolated BM neutrophils were stimulated with 3nM recombinant mouse C5a (ProSpec‐Tany TechnoGene Ltd.) in RPMI1640 culture medium containing 10% fetal bovine serum (FBS), 100 IU/ml penicillin, and 0.17 mmol/L streptomycin for 4 hours. The culture supernatant containing BM neutrophils (Neut‐CS) was used in chemotaxis assay.

### Treatment of THP‐1 or mouse monocyte with the CCR2 antagonist

Cultured THP‐1 cells (1 × 10^6^ cells) were treated with 1 ml of culture supernatant of dHL‐60 (dHL‐60‐CS) with C5a in the presence or the absence of 10 nM of the CCR2 antagonist (BMS, BMS CCR2 22; R&D Systems, Inc.) for 30 min followed by a chemotaxis assay or phalloidin stain.

For mouse primary leukocyte experiments, CD115‐positive mouse primary monocytes (1 × 10^6^ cells) were treated with 1 ml of culture supernatant of mouse BM neutrophil (Neut‐CS) with C5a in the presence or the absence of 10 nM of the CCR2 antagonist for 30 min. Mouse monocyte was used in a chemotaxis assay.

### Chemotaxis assay

The chemotaxis assay was performed using a modified Boyden chamber assay as shown in Fig. 3A [9]. 10^6^ cells of dHL‐60 cells or mouse BM neutrophils were stimulated with or without 3 nm C5a for 4 h, and the culture supernatant containing the stimulated dHL‐60 cells or mouse BM neutrophils were added to the lower chamber. 10^6^ cells of THP‐1 or CD115‐positive mouse primary monocyte stained with BCECF (2 ´,7 ´‐bis‐(2‐carboxyethyl)‐5‐(and‐6)‐carboxyfluorescein; Merck KGaA, Darmstadt) were added to a 3.0‐μm pore‐sized upper chamber (Chemotaxicell, Kurabo Industries Ltd.). After 30 min, the stained THP‐1 cells or CD115‐positive mouse primary monocytes were harvested from the lower chamber followed by cell lysis. In addition, the stained THP‐1 cells or CD115‐positive mouse primary monocytes were teated for the direct migration to C5a by adding C5a to the lower chamber. The fluorescence intensity was measured using a Perkin Elmer Plate Reader (ARVO^™^ X3 Multilabel Reader) at excitation with emission wavelengths of 485 and 535 nm, respectively. The relative chemotaxis level of THP‐1 or CD115‐positive mouse primary monocyte was calculated using the fluorescence intensity under stimulation by a dHL‐60‐CS or Neut‐CS in the absence of C5a as 1.00.

### Phalloidin stain

10^6^ cells of THP‐1 cells were seeded onto L‐lysin‐coated (Sigma‐Aldrich, Merck KGaA, Darmstadt, Germany) coverslips. The stimulated THP‐1 cells were incubated for 30 min at 37 °C and stained with Phalloidin‐iFluor 488 (Abcam plc.Cambridge, UK) and DAPI (Dojindo Molecular Technologies, Inc. Kumamoto, Japan) to capture fluorescence microscopic images (IX70, Olympus, Tokyo, Japan). The ratio of phalloidin‐positive cells to the number of DAPI‐positive cells was calculated using MetaMorph (Molecular Devices Corp, CA, USA).

### Statistical analysis

Data are expressed as the mean ± SD (standard deviation), and differences between the groups were analyzed using a two‐tailed Student’s *t*‐test or one‐way analysis of variance (ANOVA) followed by Tukey’s test or Dunnett’s test. Differences were considered to be significant at *P* < 0.05. Statistical analyses were performed using graphpad prism 5.0 (GraphPad Software, San Diego, CA, USA).

## Results

### C5a increased CCL2 secretion from neutrophil‐like dHL‐60

**Fig. 1:**
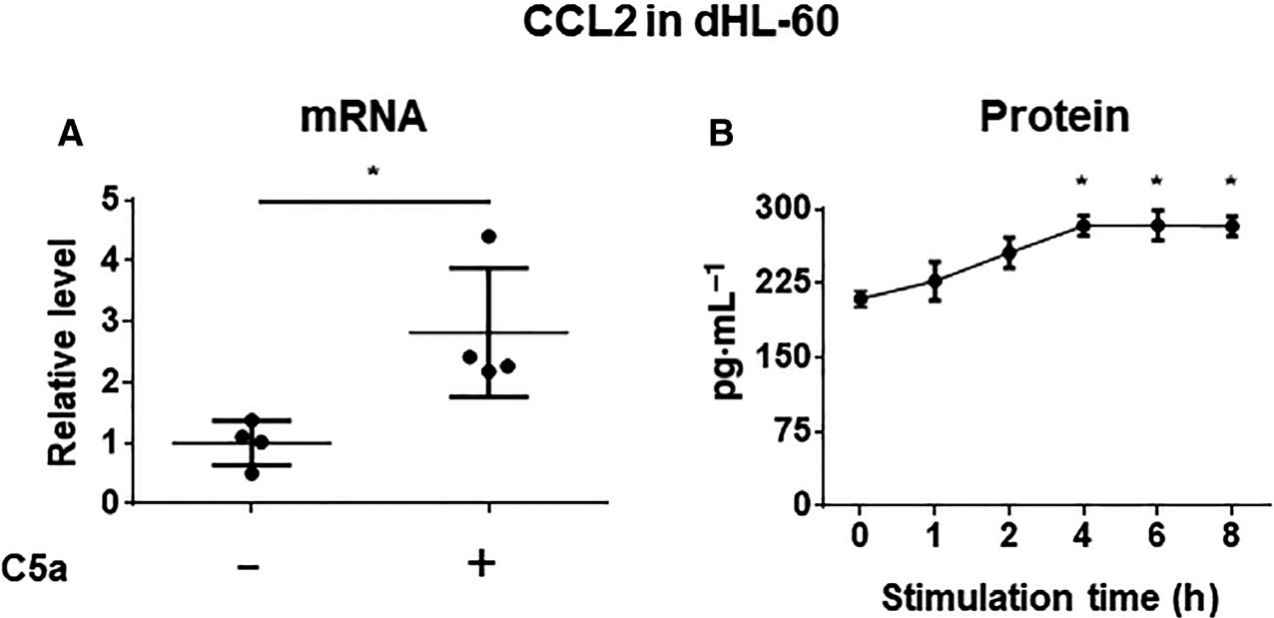
C5a upregulated CCL2 expression and secretion in neutrophil‐like dHL‐60. (A) mRNA level of CCL2 in C5a‐stimulated dHL‐60. Data presented as the mean ± SD. *P < 0.05; by two‐tailed unpaired Student’s *t*‐test. (B) CCL2 level in the culture supernatant of dHL‐60 treated with C5a was measured by ELISA in a time‐dependent manner. Data presented as the mean ± SD. *P < 0.05 by Dunnett’s test after one‐way ANOVA.

To confirm the expression level of CCL2 in neutrophil‐like differentiated HL‐60 stimulated with C5a, quantitative RT‐PCR was performed. The mRNA level of CCL2 was significantly increased in dHL‐60 treated with C5a (*P* < 0.05, *N* = 4, Fig. 1A). In addition, we performed ELISA for CCL2 in a medium derived from culturing dHL‐60 in the presence or absence of C5a to quantify the secretion from dHL‐60 by C5a. C5a significantly increased CCL2 secretion in dHL‐60 in a stimulation time‐dependent manner (0 vs. 4 h, 6 h, 8 h *P* < 0.05, *N* = 3, Fig. 1B). These results indicate that C5a induces CCL2 production in dHL‐60.

### C5a induces CCL2 expression via phosphorylation of NF‐κB in neutrophil‐like dHL‐60

**Fig. 2:**
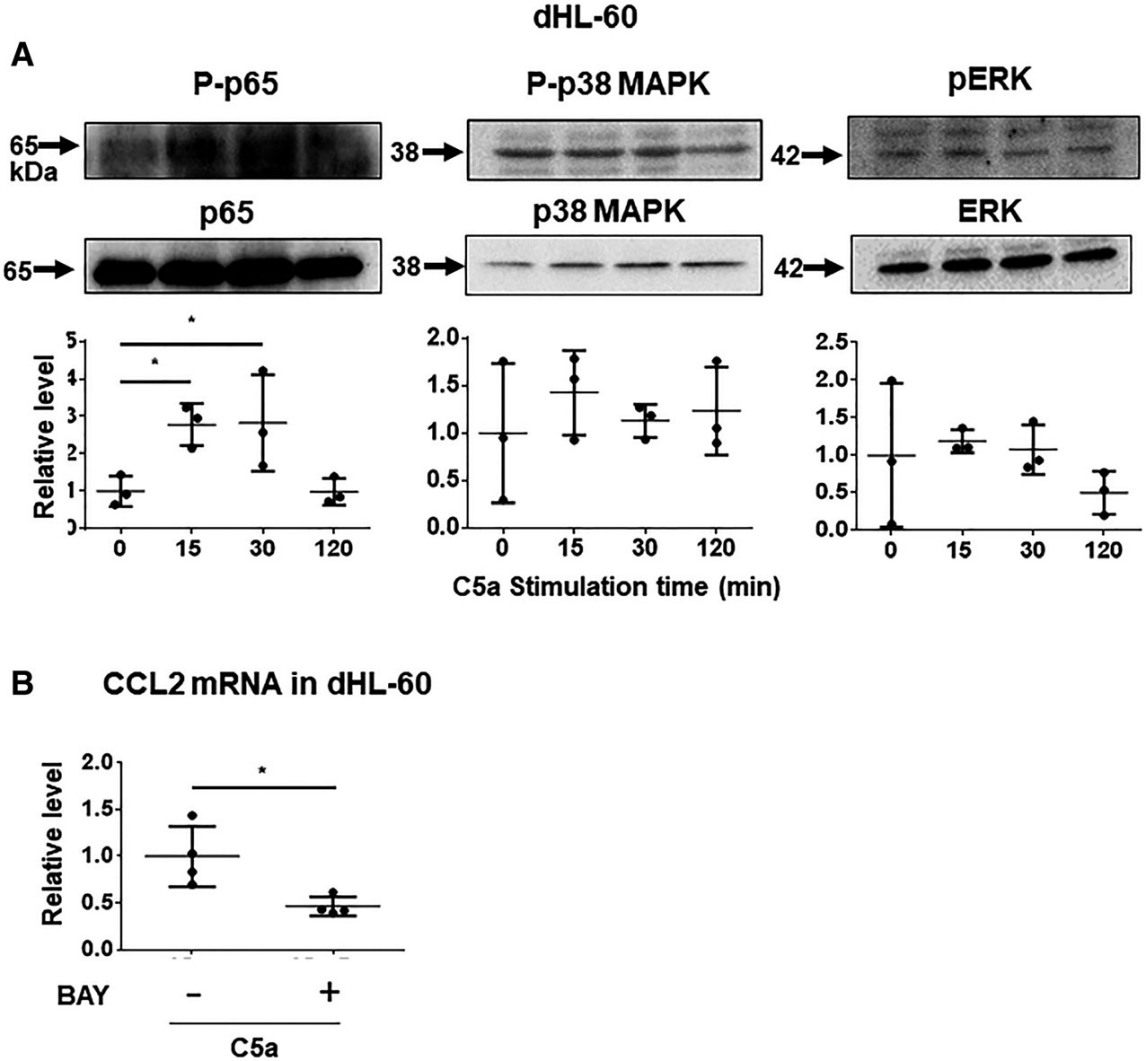
C5a induced phosphorylation of p65 NF‐κB in dHL‐60. (A) Western blot analysis for p‐p65, p‐p38, and p‐ERK in dHL‐60 treated with C5a in a time‐dependent manner. The images represent clopped membranes. The lower graph shows the relative level of phosphorylation relative to the nonphosphorylated form as one. Data presented as the mean ± SD. *P < 0.05 by Dunnett’s test after one‐way ANOVA. (B) mRNA level of CCL2 in C5a‐stimulated dHL‐60 treated with or without the IκB kinase inhibitor, BAY 11‐7082. Data presented as the mean ± SD. *P < 0.05; by two‐tailed unpaired Student’s *t*‐test.

To identify the underlying signaling pathway of CCL2 upregulation in C5a‐stimulated dHL‐60, we performed western blotting analysis for phosphorylation of p65 NF‐κB, p38 MAPK, and ERK in a time‐dependent manner. As shown in Fig. 2A and Fig. S2, phosphorylation of p65 NF‐κB—not p38 MAPK and ERK—significantly increased in C5a‐stimulated dHL‐60 cells at 15 min and 30 min after stimulation compared with 0 min (0 min vs. 15 min; *P* < 0.05, vs. 30 min; *P* < 0.05, *N* = 3 in each group). Additionally, the IκB kinase inhibitor, BAY 11‐7082, significantly suppressed CCL2 expression upregulated by C5a (*P* < 0.05, *N* = 4, Fig. 2B). These results suggested that C5a induces CCL2 expression via phosphorylation of NF‐κB in neutrophil‐like dHL‐60.

### dHL‐60‐induced CCL2 increased migration of monocytic THP‐1 and primary mouse monocytes

**Fig. 3:**
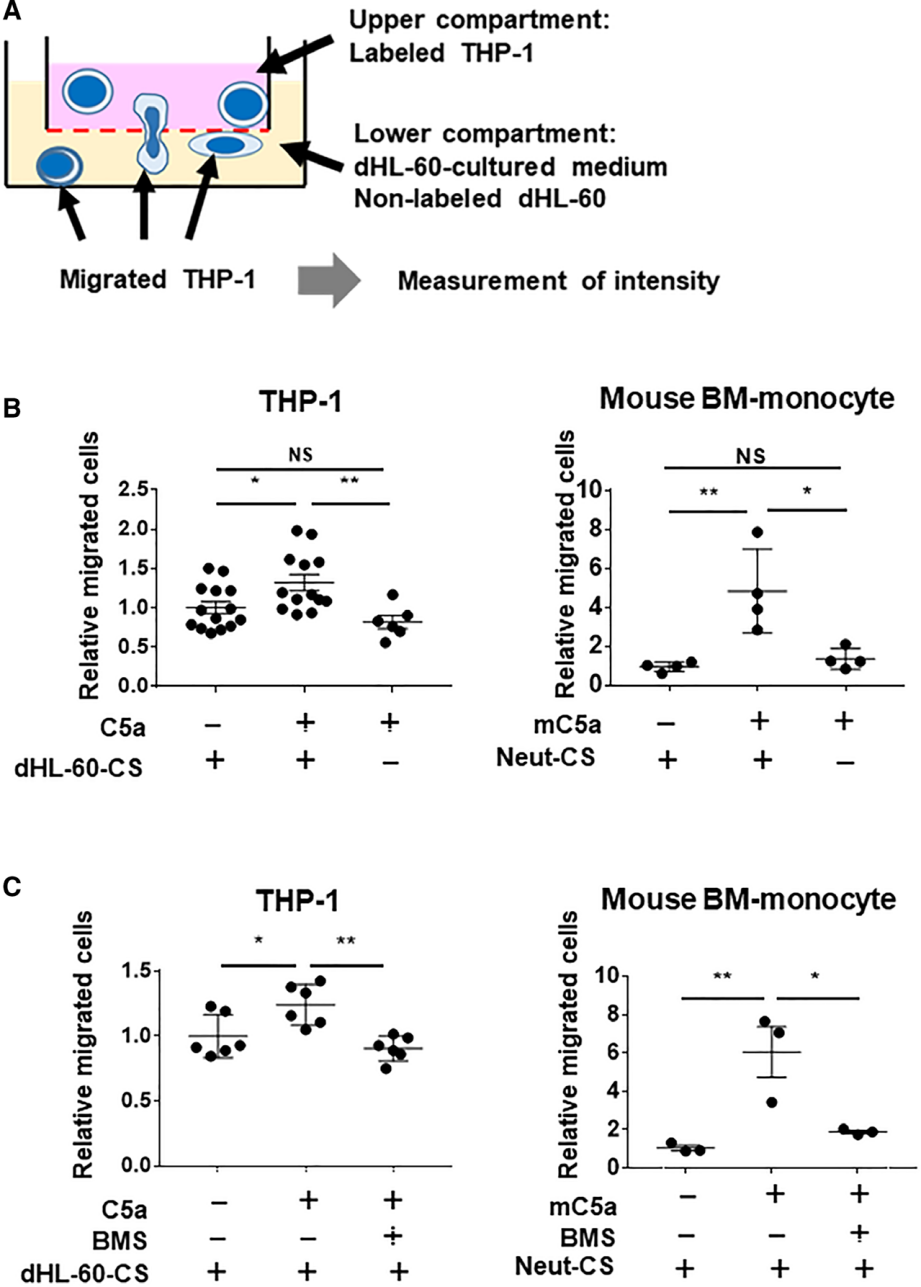
Monocytic THP‐1 cell migration by dHL‐60‐CS in the presence or absence of C5a. (A) Scheme for a method of chemotaxis assay in THP‐1 by culture supernatant of dHL‐60 (dHL‐60‐CS) in the presence or the absence of C5a. (B) Chemotaxis of THP‐1 by dHL‐60‐CS in the presence or the absence of C5a (left graph). Right graph shows chemotaxis of BM mouse primary monocyte by culture supernatant of mouse BM neutrophil (Neut‐CS) in the presence or the absence of C5a. (C) Chemotaxis of THP‐1 by dHL‐60‐CS with C5a in the presence or the absence of the CCR2 antagonist (BMS) (left graph). Right graph shows chemotaxis of BM monocyte by Neut‐CS with C5a in the presence or the absence of the CCR2 antagonist (BMS). Data presented as the mean ± SD. **P < 0.01, *P < 0.05; by Tukey’s test after one‐way ANOVA.

To investigate the involvement of neutrophil‐like dHL‐60 in the chemotaxis of monocytic THP‐1, we performed a chemotaxis assay. The dHL‐60‐CS in the presence of C5a significantly induced the chemotaxis of THP‐1 (*P* < 0.05, *N* = 14, 13; Fig. 3B). To validate the direct effect of C5a on THP‐1 and the CCL2‐CCR2 signal dependency, the chemotaxis was examined using THP‐1 stimulated with C5a directly. Direct C5a stimulation to THP‐1 did not change their chemotaxis (*N* = 6; Fig. 3B). These results indicated that the components other than C5a in the cultured medium induce migration.

Similar effect of C5a has been demonstrated using isolated primary mouse leukocytes. The culture supernatant of mouse BM neutrophil (Neut‐CS) in the presence of C5a also significantly increased the migration of CD115‐positive mouse primary monocyte (*P* < 0.01, *N* = 4; Fig. 3B), suggesting that neutrophil stimulated with C5a has the effect on monocyte migration. Furthermore, the blockage of CCL2‐CCR2 signaling in chemotaxis was examined. Treatment of the CCR2 antagonist, which blocks CCL2‐CCR2 signaling to THP‐1 or CD115‐positive mouse primary monocyte, significantly decreased upregulation of chemotaxis by dHL‐60‐CS or Neut‐CS in the presence of C5a (THP‐1; *P* < 0.01, *N* = 6, CD115‐positive mouse primary monocyte; *P* < 0.05, *N* = 3; Fig. 3C). These results suggest the involvement of CCL2 secreted from neutrophil‐like dHL‐60 or BM neutrophil stimulated by C5a, not direct C5a stimulation, in the chemotaxis of monocytic THP‐1 or monocyte.

### dHL‐60‐CS in the presence of C5a induced actin polymerization in THP‐1 cells

**Fig. 4:**
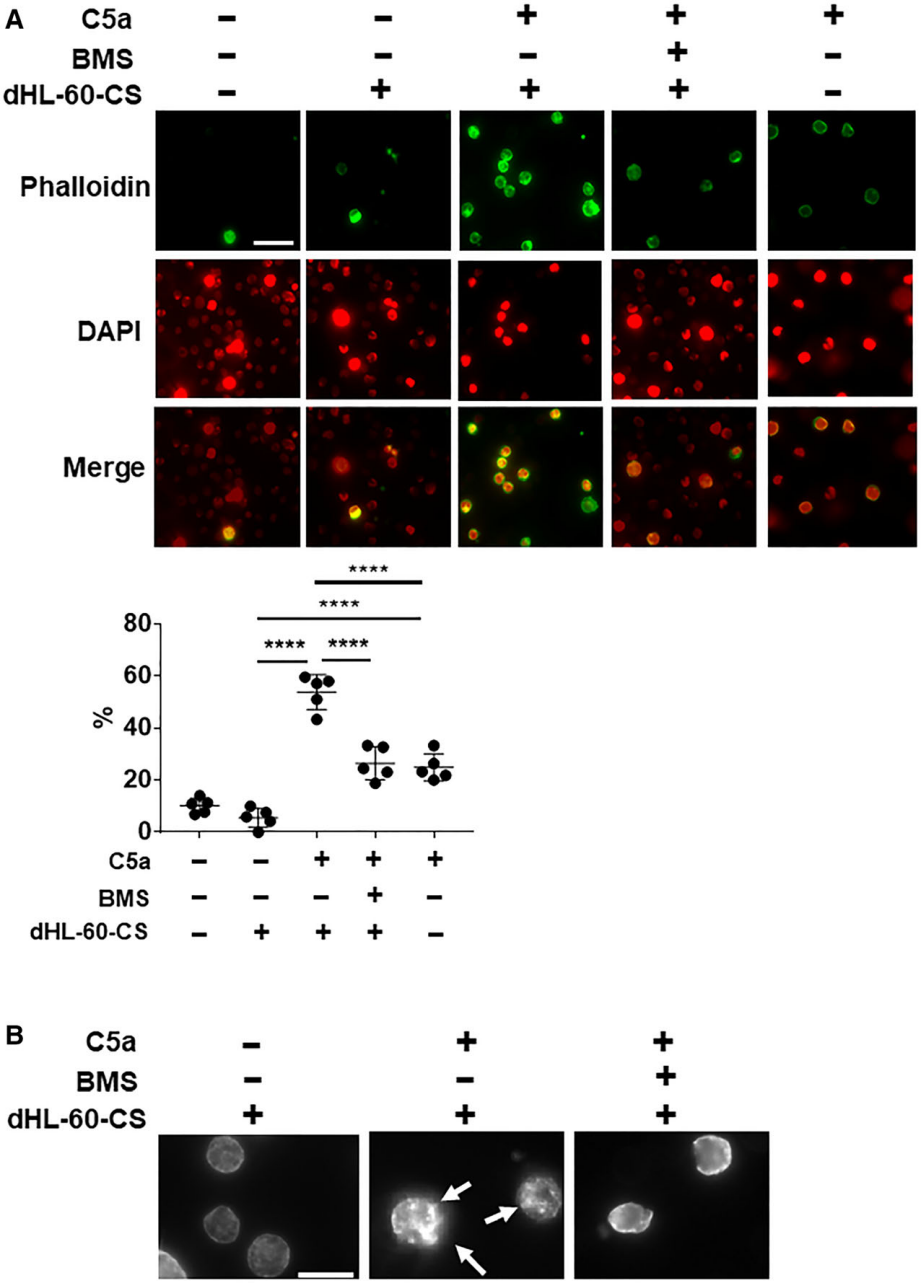
Phalloidin stain for THP‐1 treated with dHL‐60‐CS in the presence or absence of C5a with or without CCR2 antagonist. (A) Images of phalloidin stain for THP‐1 treated with C5a, culture supernatant of dHL‐60 (dHL‐60‐CS) in the presence or the absence of C5a, and dHL‐60‐CS with C5a in the presence of CCR2 antagonist (BMS). Green indicates phalloidin, and red indicates DAPI. Magnification of objective is 40×. Graph shows quantification of phalloidin‐positive cells. Bar: 50 µm. Data presented as the mean ± SD. ****P < 0.0001 by Tukey’s test after one‐way ANOVA. (B) Representative images show the aggregation of actin polymerization by phalloidin stain for THP‐1 treated with dHL‐60‐CS with C5a in the presence or the absence of the CCR2 antagonist (BMS). Arrows indicate the aggregation of actin. Bar: 20 µm.

Leukocyte migration depends on intracellular cytoskeleton [10]. In particular, actin network plays a dominant role in cellular cytoskeletal architecture [11]. Hence, we examined actin polymerization in THP‐1 under the dHL‐60‐CS in the presence or the absence of C5a. The number of phalloidin‐positive THP‐1 significantly increased under the stimulation with dHL‐60‐CS in the presence of C5a in comparison with the absence of C5a (*P* < 0.0001, *N* = 5; Fig. 4A). Though direct C5a stimulation to THP‐1 significantly increased the ratio of phalloidin‐positive cells compared with the dHL‐60‐CS without C5a, the ratio was significantly lower than dHL‐60‐CS with C5a. These results suggested that the components other than C5a in the cultured medium have stronger effect on actin polymerization. Moreover, the addition of the CCR2 antagonist significantly decreased the number of phalloidin‐positive THP‐1 (*P* < 0.0001, *N* = 5; Fig. 4A). Individual observation of THP‐1 revealed that the intense aggregation of phalloidin was induced after stimulation with dHL‐60‐CS in the presence of C5a. However, this phenomenon was diminished by the treatment of CCR2 antagonist as shown in Fig. 4B. These results indicate that dHL‐60‐CS in the presence of C5a induces actin polymerization via CCL2‐CCR2 signaling.

## Discussion

Atherosclerosis is a pro‐inflammatory disease associated with leukocyte adhesion and migration by accumulation of lipid in the vessel wall [12, 13]. Previous pathological examinations revealed that the majority of infiltrated leukocytes are monocytes or macrophages [12, 14]. In contrast, we were able to document that neutrophils interact with the vessel wall in HFD‐fed mice [3]. Therefore, we hypothesized that vascular inflammation shifts from an acute to chronic phase by monocyte migration, which is dependent on neutrophils. Moreover, CCL2 expression in neutrophils and the CCL2 level in the blood of HFD‐fed mice increased compared with those in mice fed a normal diet [3]. In addition, CCL2 is the chemoattractant factor for monocyte [15]. Therefore, these findings indicate that CCL2 secretion from neutrophil stimulated with C5a induces monocyte migration. Hence, we focused on crosstalk between neutrophils and monocytes via CCL2.

We clarified that neutrophil‐like dHL‐60 stimulated with C5a induces the migration of monocytic THP‐1. This finding was confirmed using isolated mouse primary neutrophils and monocytes. (Fig. 3B). These results indicate that neutrophil has a role in monocyte migration. Meanwhile, direct stimulation of C5a to THP‐1 or monocyte did not change their migration, suggesting that the direct effect of C5a to THP‐1 plays minimum role in this phenomenon. Considered in combination with our previous report that C5a induces neutrophil adhesion to vessels in mice, CCL2 secreted from adhered neutrophil on the vessel may lead to monocyte migration to the vessel depending on the concentration gradient of CCL2 in the vessel wall.

Our data suggest that cytoskeleton reorganizations through CCL2‐CCR2 axis play a key role in monocytic THP‐1 or monocyte migration [16, 17], as was previously observed [18, 19]. As shown in Fig. 4A, dHL‐60‐CS in the presence of C5a induced actin polymerization. Furthermore, the CCR2 antagonist inhibited the enhanced action of polymerization in THP‐1, which suggests that neutrophil contributes to actin polymerization in monocyte through a secretion of the CCL2 from neutrophils. Leukocytes migrate through the formation of projection on the cell surface and the actin polymerization, which generate the force for crawling [20]. As shown in Fig. 4B, the aggregation of actin polymerization was observed in THP‐1 stimulated with dHL‐60‐CS in the presence of C5a. Taken together, these results suggested that neutrophil may induce monocyte migration through the induction of actin rearrangement by CCL2‐CCR2 signaling.

C5a‐C5a receptor signaling enhanced the IL‐8 promoter and activated NF‐κB in peripheral blood mononuclear cells and murine RAW264.7 macrophage cells [21]. Infection activated MAPK signaling through C5a upregulation [22]. In addition, CCL2 expression is regulated by NF‐κB binding to its promoter site [23]. Hence, we examined the signaling pathway for CCL2 expression in neutrophil‐like dHL‐60 stimulated by C5a. C5a induced phosphorylation of p65 NF‐κB, and the inhibition downregulated CCL2 expression by C5a in these cells (Fig. 2B). Therefore, these results suggest that the activation of the CCL2 promoter by phosphorylation of NF‐κB leads to increased CCL2 secretion from neutrophils. Moreover, the enhancement of CCL2 secretion from neutrophils may contribute to the higher plasma CCL2 level in subjects with acute coronary syndromes [24].

In conclusion, we found that neutrophil‐like dHL‐60 stimulated with C5a induces the migration of monocytic THP‐1 through phosphorylation of p65 NF‐κB and that dHL‐60‐derived CCL2 plays an important role in the crosstalk between these cells. These results suggest that there may be a transition process from acute to chronic inflammation in atherosclerotic vascular inflammation.

## Conflict of interest

The authors declare no conflict of interest.

## Author contributions

SMRD, MO, and MY wrote the article; SMRD performed quantitative RT‐PCR, western blotting, and statistical analysis; MO performed phalloidin stain, chemotaxis, and statistical analysis; and MD performed chemotaxis assay; MO decided conception, design, and methodology; and MY was supervisor.

## Data availability statement

The data that support the findings of this study are available from the corresponding author [masa.vasc@tmd.ac.jp] upon reasonable request.

## Supporting information

Fig. S1. Character of isolated cells from bone marrow derived leukocytes by magnetic beads.
**Fig. S2.** Full images of western blotting for p‐p65/p65, p‐p38/p38 and pERK/ERK.
