# Supplementary material for: Complement C5a‐triggered differentiated HL‐60 stimulates migration of THP‐1 monocytic leukocytes via secretion of CCL2
Source: FEBS Open Bio. 2021 Apr 3;11(5):1374–81. doi: 10.1002/2211-5463.13144 (PMC8091577; doi:10.1002/2211-5463.13144)
Supplement: Supplementary file 1 — Fig. S1. Character of isolated cells from bone marrow derived leukocytes by magnetic beads. Fig. S2. Full images of western blotting for p‐p65/p65, p‐p38/p38 and pERK/ERK. [file FEB4-11-1374-s001.pdf]

Supplementary materials

Complement C5a-triggered differentiated HL-60 stimulates migration of THP-1 monocytic leukocytes via secretion of CCL2

Syed Masudur Rahman Dewan[1], Mizuko Osaka[1,2], Michiyo Deushi[1], Masayuki Yoshida[1]

1Department of Life sciences and Bioethics, Graduate School of Medical and Dental Sciences, Tokyo Medical and Dental University, Tokyo, Japan
2Department of Nutrition in Cardiovascular Disease, Graduate School of Medical and Dental Sciences, Tokyo Medical and Dental University, Tokyo, Japan

Address correspondence to:
Masayuki Yoshida, MD
Department of Life Sciences and Bioethics, Graduate School of Medical and Dental Sciences,
Tokyo Medical and Dental University
1-5-45, Yushima, Bunkyo-ku, Tokyo 113-8519, Japan.
Tel: +81-3-5803-4617.
Fax: +81-3-5800-3380.
E-mail: masa.vasc@tmd.ac.jp

Supplementary Fig. 1

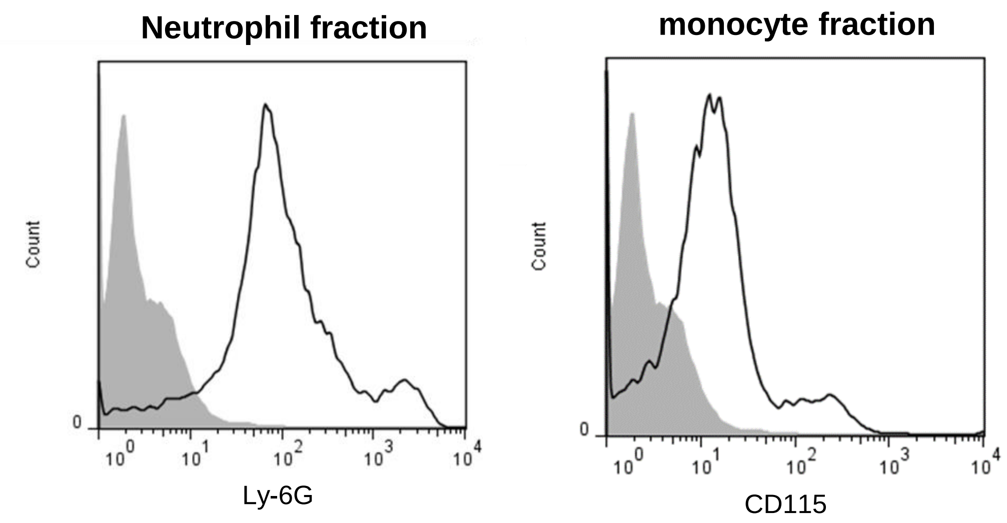

Supplementary Fig. 1 Character of isolated cells from bone marrow derived leukocytes by magnetic beads.
Neutrophil and monocyte fraction isolated from bone marrow were validated each character by flowcytometery. Gray is indicated control IgG.

Supplementary Fig. 2

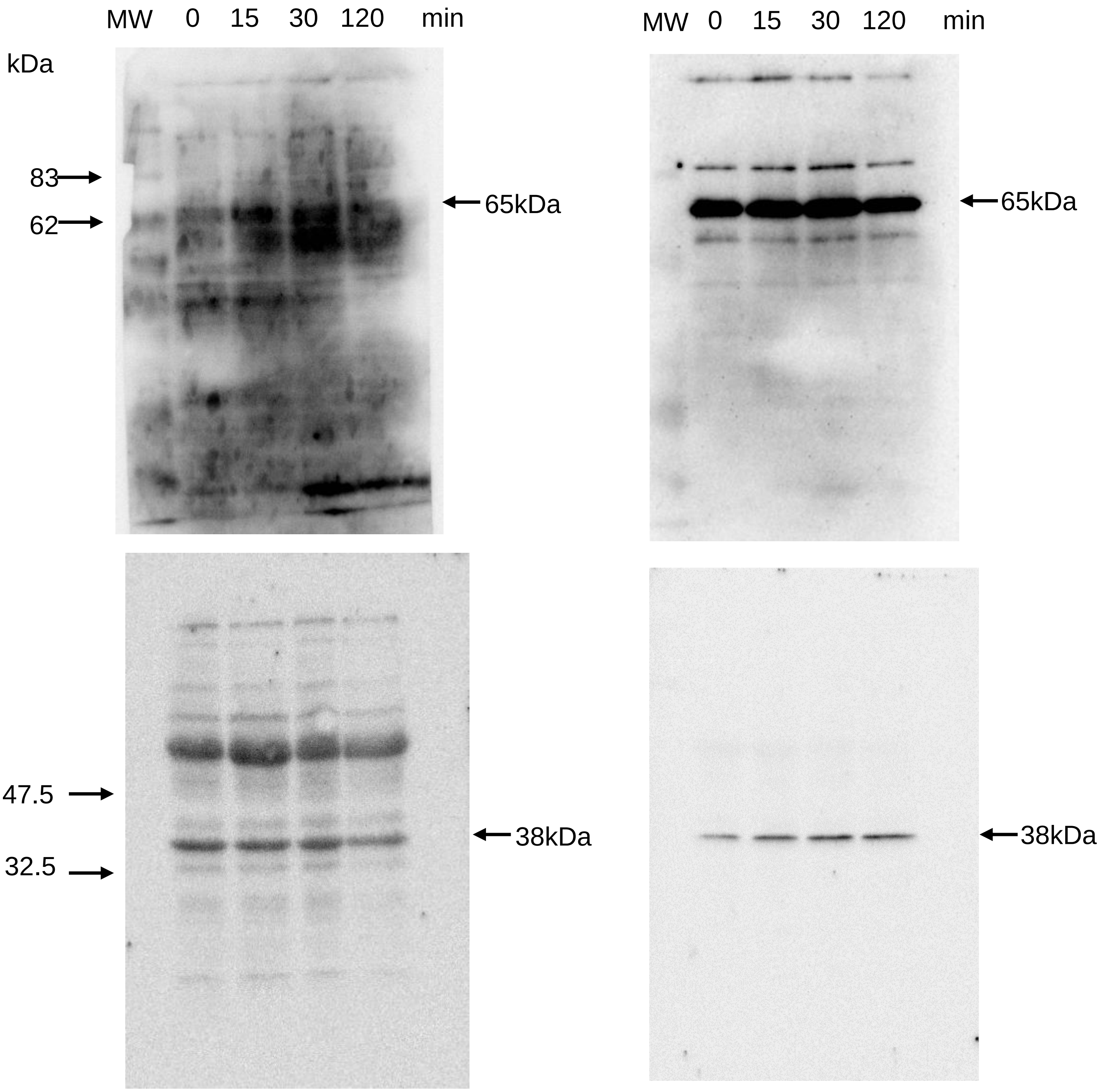

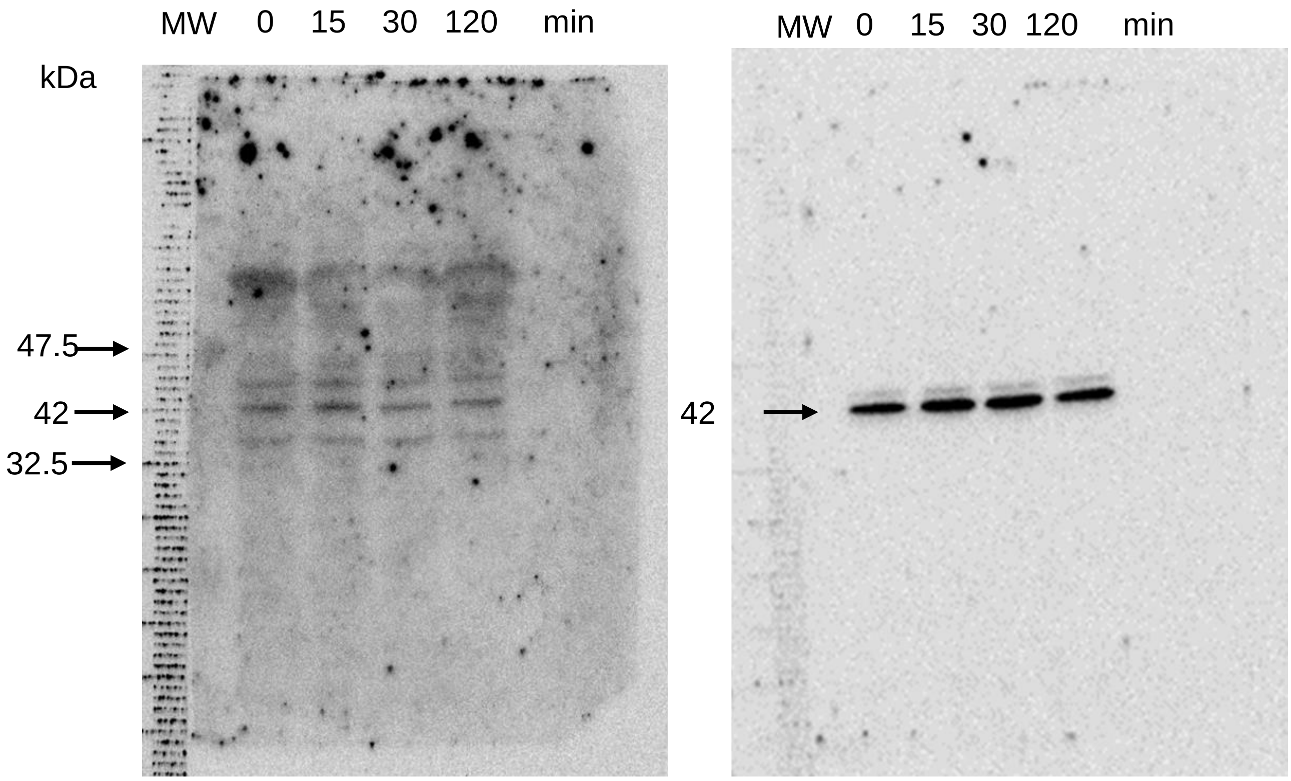

Supplementary Fig. 2 Full images of western blotting for p-p65/p65, p-p38/p38 and pERK/ERK. Western blot analysis for p-p65, p-p38, and p-ERK in dHL-60 treated with C5a were performed in a time-dependent manner. Cropped these representative full images showed in Figure 2A. Arrow heads indicated each molecular weight.
